# Efficient federated learning via aggregation of base models

**DOI:** 10.1371/journal.pone.0327883

**Published:** 2025-08-14

**Authors:** Pan Wang, Zhengyi Zhong, Ji Wang

**Affiliations:** Laboratory for Big Data and Decision, National University of Defense Technology, Changsha, Hunan, China; TTU: Tan Tao University, VIET NAM

## Abstract

*Federated Learning* (FL), as a distributed computing framework for training machine learning (ML) models, has garnered significant attention for its superior privacy protection. In typical FL, a subset of client models is randomly selected for aggregation in each iteration, which performs well when the data is independent and identically distributed (IID). However, in real-world scenarios, data is often non-independent and identically distributed (Non-IID). Random selection cannot capture knowledge from different data distributions, resulting in a global model with lower accuracy and slower convergence. To address this challenge, we propose *base models*, which are models with diverse data distributions on clients. By combining the parameters of these base models, we can approximate all client models. Meanwhile, we sufficiently demonstrate *the existence of base models*. Then we employ *the evolutionary algorithm* (EA) to identify base models on distributed clients by encoding client IDs and optimizing client selection through crossover, mutation, and other evolutionary operations. Our method addresses the issue of low efficiency in random selection. We conduct experiments on the FashionMNIST, MNIST, and TodayNews datasets, applying the proposed method to FL frameworks such as FedAvg, FedProx, and SCAFFOLD, all of which show superior performance and faster convergence.

## 1 Introduction

**Fig 1:**
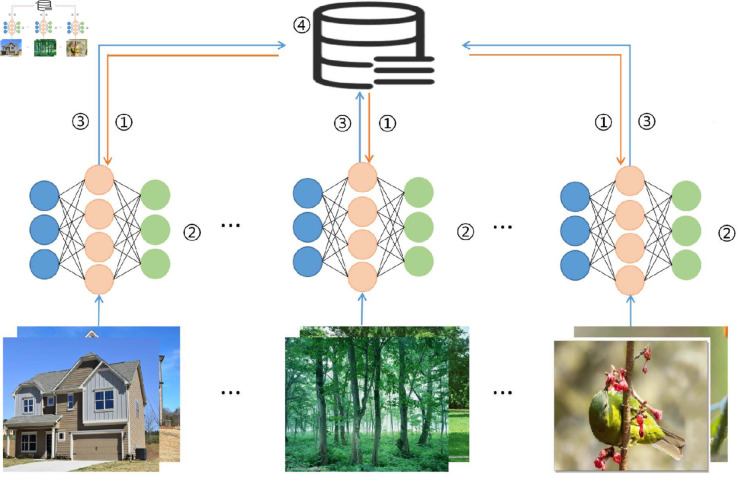
The process of FL. Step 1: Server distributes the global model; Step 2: Clients train locally; Step 3: Clients upload the local model; Step 4: Server aggregates models.

With the rapid advancement of technologies such as the Internet of Things (IoT) and artificial intelligence (AI), mobile devices like smartphones and tablets are increasingly capable of storing, processing, and transmitting vast amounts of data. However, due to privacy concerns and the enforcement of strict data protection regulations [1], internet companies are unable to gather clients data for model training. To tackle this real-world challenge, McMahan *et al*. [2] proposed Federated Learning (FL), which aims to facilitate client model training among multiple participants while safeguarding data privacy. As a distributed computing framework, FL [2] can aggregate model knowledge from different clients by only uploading model parameters. Ultimately obtains a global model with diverse data features. FL involves four processes. Initially, the server distributes the global model to the clients. Secondly, the clients utilize their local data to train and obtain client-side models. Subsequently, the clients update their local models and upload them to the server. Finally, the server aggregates the client models to obtain the global model. This iterative process continues until the model converges or achieves the desired performance. The specific process is illustrated in Fig 1.

Classical FL perform well under IID settings but exhibit poor performance under Non-IID settings. This is because the non-independent and identically distributed (non-i.i.d.) data on the clients may lead to similar data distributions among different clients. Traditional methods, which select clients for aggregation randomly, are likely to include clients with similar distributions. As a result, the global model needs to undergo more rounds of aggregation to capture the full knowledge from the clients, ultimately leading to a decrease in both the convergence speed and accuracy of the global model. Researchers combined FL with other sophisticated methods, such as regularization [3,4], knowledge distillation [5–7], and client selection [8,9], to tackle the challenges posed by data heterogeneity in FL. For example, Chai *et al*. [10] categorized clients into different layers for stratified training, while Zhu *et al*. [5] applied data-free distillation methods to mitigate the effects of data heterogeneity. While these approaches are adept at addressing the issue of data heterogeneity, several critical issues remain unresolved. In typical FL algorithms, server randomly selects models for aggregation, then using the aggregation of these “lucky” clients as the global model. However, models selected through this method are often biased, since the characteristics of the model directly reflect the characteristics of the data, and data distributions of randomly selected clients are likely to exhibit high similarity. Hence, it is difficult to incorporate the diverse data characteristics into the training process.

Marfoq *et al*. [11] proposed and demonstrated that that the data distribution of each client is a mixture of *M* latent distributions. This finding underscores the inherent diversity of client data and suggests that in the model aggregation process of federated learning, randomly selecting clients may lead to the aggregation of models with similar data distributions, potentially failing to capture the full spectrum of data diversity. This motivates us to propose the concept of base models, which refer to a set of client models with the most distinct data distribution differences, correspond to local data distributions that exhibit orthogonality. Via linear parameter combinations, these models can cover all clients’ feature spaces and approximate any client model. On distributed clients, there always exist *N* base models that can represent any model through the combination of parameters. This approach enables the global model to integrate features from base models as much as possible. In other words, we can select client models that are as orthogonal as possible to the global model for aggregation. This helps the global model incorporate knowledge from client models with significantly different data distributions, thereby accelerating the convergence speed and accuracy of the model. This approach is orthogonal to previous methods. In large-scale client scenarios, the identification of base models is challenging. The genetic algorithm is particularly suitable for global search through crossover, mutation, and other operations. Therefore, this paper using the genetic algorithm to solve for the base models on clients. Assuming a fixed number of clients selected in each round, the best-performing base models are chosen from all clients for aggregation, thereby accelerating the FL process and enhancing performance.

The key contributions of this paper are as follows:

- Introducing the concept of base models under the FL architecture, and theoretically proves the existence of base models, which can enhance the aggregation effect of models, and accelerate the learning progress.
- Proposing an efficient federated learning method via aggregation of base models, utilizing the genetic algorithm to optimize the selection of base models, thereby globally optimizing the iterative process of FL.
- We conduct experiments on image and text datasets, including FashionMNIST, MNIST and TodayNews, using various classic FL frameworks such as FedAvg [2], FedProx [3], and SCAFFOLD [12]. Our method shows superior performance compared to related approaches.

The rest of the paper is organized as follows. Sect 2 reviews the advancements in federated learning, heterogeneous federated learning, and client selection. Sect 3 analyzes the existence of base models and proves relevant lemmas. Sect 4 details the process for solving base models and provides algorithm. Sect 5 validates the proposed method, with experimental results demonstrating its superiority. Sect 6 discusses our research and highlights the limitations of the study. Sect 7 summarizes the paper.

## 2 Related work

### 2.1 Federated learning

In 2016, Google proposed FL [2] as a distributed learning framework, which aggregates models from distributed clients without the need to upload user data, thereby safeguarding user privacy. With the rapid development of the Internet of Things, FL has garnered significant attention in various fields such as next-word prediction on keyboards [13], financial fraud detection [14], and medical impact analysis [15]. Current research on FL primarily focus on several challenges: communication bottlenecks, client system heterogeneity, privacy protection and data heterogeneity [16].

Firstly, the need for communication between clients and the server in FL gives rise to communication bottlenecks. Wireless communication, which typically has lower bandwidth and speed compared to wired communication, becomes a limiting factor for FL. To address this issue, researchers have explored a number of techniques, These include reducing communication frequency by communicating only once or at specific intervals [17–19], employing quantization methods to decrease the size of communication vectors [20–22], and utilizing sparsification techniques to randomly sparsify the gradients of local training models, thereby reducing communication costs [23–25].

Secondly, in the distributed computing framework of FL, different clients likely exhibit significant differences in memory and computation, leading to the exclusion of resource-constrained clients from training and posing challenges of system heterogeneity. Research [9] explored an optimized method for client selection to address heterogeneity issues. Furthermore, Li *et al*. [3] proposed the FedProx, which mitigates heterogeneous issues by improving client loss through the addition of regularization terms in the loss function. This work has garnered significant attention.

Thirdly, preserving privacy [26,27] presents a significant challenge in the field of federated learning. Jagarlamudi *et al*. [28] conduct a comprehensive survey on privacy measurement within federated learning, identifying current gaps and suggesting future directions, including the integration of quantum computing and other cutting-edge technologies. Rabieinejad *et al*. [29] propose a two-level privacy-preserving framework that combines federated learning with partially homomorphic encryption to enhance security and reduce attack prediction errors. Meanwhile, Yazdinejad *et al*. [30] concentrate on reinforcing privacy and security in CIoT devices through federated learning and innovative encryption techniques. Collectively, these methods have propelled the advancement of privacy protection within federated learning. Furthermore, the privacy protection of federated learning in practical applications [31,32] remains to be further explored.

Furthermore, apart from hardware disparities, distributed clients may also face significant differences in local data, resulting in data heterogeneity challenges. A study by Zhao *et al*. [33] explored methods to improve accuracy under non-IID settings and demonstrated that weight discrepancies during training are a key factor contributing to accuracy degradation. Systems like Astraea [34] has been employed to address global imbalanced data problems by selectively combining biased local data to create more balanced datasets, and has implemented z-score based data augmentation methods to alleviate global data imbalances. Additionally, algorithms such as Tucker [35] decomposition-based algorithms have been proposed to fuse heterogeneous data in FL. We systematically introduce the challenges faced by FL and summarize related work. Next, we will conduct further research on data heterogeneity.

### 2.2 Data heterogeneity in federated learning

Data heterogeneity in FL refers to the inconsistencies in data distribution among clients, where samples are non-independent and identically distributed (Non-IID). In FL, federated aggregation methods play a crucial role in updating the global model, which are utilized to aggregate parameters from various participants (such as tablets and smartphones) and update the global model, ultimately determining the success of model training [36–40].

McMahan *et al*. [2] proposed the federated averaging algorithm, which computes the average of received parameters to update the global model, then returned to clients for further training. Wang *et al*. [41] introduced the trimmed mean aggregation method, which clips model updates within a predefined range. This approach helps reduce the impact of outliers and potential malicious updates from clients on the results. Reyes *et al*. [42] presented the federated weighted aggregation method, where the server weights the contribution of each client in the global model based on client performance. Liu *et al*. [43] introduced a hierarchical aggregation method that involves conducting local aggregation at lower levels of the hierarchy before transmitting the results to higher levels, improving the convergence efficiency of the global model.

The aforementioned FL algorithms have shown preferable results in the client heterogeneity. However, in scenarios such as network congestion and limited communication resources, clients often face upload delays, packet loss, and other challenges. Literature [44–49] has applied exponential moving average algorithms to FL, achieving effective parameter transmission and model updates even under constrained communication resources. To address issues faced with an increasing number of clients, some researchers have investigated resource allocation method [50] in wireless networks for FL. While these methods effectively address challenges related to an increasing number of clients, randomly selected models are often trained on data from similar distributions, resulting in slower convergence and reduced accuracy.

### 2.3 Client selection

Due to the Non-IID local data on clients, there is a significant deviation between the local optimization objectives and the global optimization objective. Randomly selected clients for aggregation during the FL process will exacerbate the negative impact of data heterogeneity. Fu *et al*. [51] highlighted that client selection in FL is an emerging topic, and an effective client selection scheme can significantly improve model efficiency. Chai *et al*. [10] confirmed the detrimental effects of random client selection on federated learning performance through theoretical analysis and experimental validation. Literature [8] introduces the Oort to establish data selection criteria to obtain clients with more informative and fast-executing training capabilities. Literature [9] suggested that in heterogeneous environments, prioritizing clients with higher local loss values can accelerate the convergence speed of the global model, thereby improving communication efficiency and providing the proof of convergence for biased client selection in FL.

Additionally, Jin *et al*. [52] selected appropriate clients and excluded unnecessary model updates to save resources, designing an online learning algorithm that jointly controls participant selection in an online manner. Ribero *et al*. [53] proposed a selection strategy based on client availability, progressively minimizing the impact of client sampling variance on the convergence of the global model and thereby enhancing federated learning performance. Luo *et al*. [54] proposed an adaptive client sampling algorithm to address system and statistical heterogeneity, minimizing convergence time. Marnissi *et al*. [55] designed a device selection strategy based on the importance of gradient norms. AdaFL [56] dynamically adjusts the number of selected clients using a piecewise function, starting with a smaller selection size to reduce communication overhead, and gradually increasing the selection size to enhance the model’s generalization capabilities. TiFLCS-MAR [57] is integrated into the federated learning framework, allowing for a comprehensive evaluation of client attributes and employing a tiered strategy to mitigate issues arising from client heterogeneity. These methods prioritize aggregating clients with large amounts of data, potentially excluding clients with small amounts of data from participating in the aggregation process. However, these underrepresented clients may have distinct data distributions that are orthogonal to those of other client models. This paper further optimizes client selection by the evolutionary algorithm (EA), aiming to aggregate base models with lricher data distributions, accelerating the convergence speed and improving the accuracy of the global model.

## 3 Theoretical analysis

In contrast to the random selection of clients in traditional FL, we propose an aggregation method for FL via the aggregation of base models to enhance model training efficiency. Building on the concept of base distribution introduced by Marfoq *et al*. [11], this section theoretically proves the existence of base models on distributed clients.

Assuming there exists a model $s_{c}\in S$ within the client set *C* that performs task classification, each model can be represented as a weighted average combination of *N* base models $s_{\theta_{n}^{\prime \prime }}$, this meaning the local data distribution *D*_*c*_ corresponding to the client model can be expressed as a weighted average combination, where 1≤n≤N. The data on client c∈C is generated according to the data distribution $D_{𝔠}$ within the range of X×Y, where X×Y represents the Cartesian product of the input space *X* and the output space *Y*. The data distribution ${\left\{D_{c}\right\}}_{c\in C}$ on the clients *C* is generally different, leading to different models *s*_*c*_ trained by the clients. Based on the above conditions, the following optimization problem is considered:

$$\forall c\in C,\underset{s\in S}{minimize}L_{D_{c}}\left(s_{c}\right),$$ (1)

thus, the objective is to minimize the loss during training the model on the local data distribution, where $l:\Delta^{\left|Y\right|}\;\times \;Y\mapsto {\mathbb{R}}^{+}$ is the loss function, and $s_{c}:X\mapsto \Delta^{\left|Y\right|}$ is the client model mapping from the input space *X* to the output space *Y* (where $\Delta^{\left|Y\right|}$ denotes the unitary simplex of dimension |Y|. The evaluation of the loss for the model *s*_*c*_ during training is defined by $L_{D_{c}}(s_{c})=E_{(x,y)\sim D_{c}}[l(s_{c}(x),y)]$. For any (x,y)∈X×Y, we denote the joint distribution density related to *D*_*c*_ as *p*_*c*_(*x*,*y*), and the marginal densities as *p*_*c*_(*x*) and *p*_*c*_(*y*).

During the training process, an initial screening of base models is conducted, and the corresponding clients are added to the set $[C_{\sigma }]\overset{\Delta }{=}\{1,2,\ldots ,C_{\sigma }\}\subseteq C$ for aggregation. Subsequently, through multiple rounds of iteration, more suitable base models are gradually selected. $H_{c}=\{h_{c}^{\left(i\right)}=(x_{c}^{\left(i\right)},y_{c}^{\left(i\right)}){\}}_{i=1}^{m_{c}}$ represents the dataset extracted independent and identically from the data distribution *D*_*c*_ on client $c\in [C_{\sigma }]$, with $m=\sum_{c=1}^{C}m_{c}$ denoting the total dataset size. The purpose of selecting and aggregating base models is to find client models with strong diversity and low similarity, aiming to reduce the loss $L_{D_{c}}$ and enhance the model’s generalization ability. Marfoq *et al*. [11] point out that without additional assumptions on the local distribution *p*_*t*_(*x*,*y*), the improvements from collaboration among clients via federated learning (FL) algorithms are limited. This collaboration may only result in an increase of a constant factor in sample complexity, rather than a significant reduction in the required sample size. Assuming that there exists some structural relationship (such as similarity or correlation) between the output distributions *p*_*t*_(*y*|*x*) across different clients would enable FL algorithms to share information and learn more effectively, thereby significantly reducing sample complexity. Based on these considerations, the following assumptions are made.

**Assumption 1.** *There exist n base models $s_{\theta_{n}^{\prime \prime }}$ with corresponding data distributions ${\tilde{D}}_{n},1\leq n\leq N$. When c∈C, the client model *s**_*c*_ *is a combination of the base models ${\left\{s_{\theta_{n}^{\prime \prime }}\right\}}_{n=1,2\ldots ,N}$, with corresponding weights $\gamma_{c}^{\prime \prime }=[\gamma_{c1}^{\prime \prime },\ldots ,\gamma_{cN}^{\prime \prime }]\in \Delta^{N}$. It can be expressed as:*

$$z_{c}\sim N(\gamma_{c}^{\prime \prime }),((x_{c},y_{c})|z_{c}=n)\sim {\tilde{D}}_{n},\forall c\in C,$$ (2)

φ(γ) is a polynomial distribution parameterized by *γ*, and the probability density distributions related to ${\tilde{D}}_{n}$ are denoted as *p*_*n*_(*x*,*y*), *p*_*n*_(*x*), and *p*_*n*_(*y*).

**Assumption 2.** *For any n∈[N], it holds that*

$$p_{n}(x)=p(x),$$ (3)

Assumption 2 implies that we can proceed with the inference in discriminative and classification models (such as neural networks). More specifically, we consider a set of trained classification models $\tilde{S}$ for the next steps.

**Assumption 3.** $\tilde{S}={\left\{s_{\theta }\right\}}_{\theta \in {\mathbb{R}}^{d}}$* is a set of base models parameterized by $\theta \in {\mathbb{R}}^{d}$ after local training, with the boundary of this set contained within S. For any base model $s_{\theta_{n}^{\prime \prime }}$ in $\tilde{S}$ when n∈[N], its corresponding data distribution is ${\tilde{D}}_{n}$, such that:*

$$l(s_{\theta_{n}^{\prime \prime }}(x),y)=-\log p_{n}(y|x)+a,$$ (4)

where a∈ℝ is a normalization constant, and the function l(·,·) is the log loss function of *p*_*n*_(*y*|*x*). The models in $\tilde{S}$ are base models, denoted by $\theta^{\prime \prime }\in {\mathbb{R}}^{N\;\times \;d}$ representing a matrix where the *n*-th row is $\theta_{n}^{\prime \prime }$, and $\Gamma^{\prime \prime }\in \Delta^{C\;\times \;N}$ representing a matrix where the *c*-th row is $\gamma_{c}^{\prime }\in \Delta^{N}$. Similarly, θ and Γ can represent any parameters in the matrix.

Under the aforementioned Assumptions, it is known that *p*_*c*_(*x*,*y*) is determined by $\theta^{\prime \prime }\in {\mathbb{R}}^{N\;\times \;d}$ and $\gamma_{c}^{\prime \prime }\in \Delta^{N}$. We can prove that the optimal local model $s_{c}^{\prime \prime }\in S$ on client c∈C can be represented as a weighted average of the base models in the set $\tilde{S}$.

**Proposition.** *Using l(·,·) to represent mean squared error loss, regression loss, or cross-entropy loss, let $\hat{\theta }$ and $\hat{\Gamma }$ be the solutions to the following optimization problem:*

$$\underset{\theta ,\Gamma }{minimize}\underset{c\sim D_{c}}{E}\underset{(x,y)\sim D_{c}}{E}\left[-\log p_{c}(x,y|\theta ,\gamma_{c})\right].$$ (5)

For any client model ${s_{c}^{\prime }}^{\prime }$ under the distribution *D*_*c*_, Under Assumptions 1, 2, and 3, client model can be represented as:

$${s_{c}^{\prime }}^{\prime }=\sum_{n=1}^{N}{\hat{\gamma }}_{cn}s_{{\hat{\theta }}_{n}}(x),\;\forall c\in C,$$ (6)

by minimizing $E_{(x,y)\sim D_{c}}[l(s_{c}(x),y)]$, problem 1 can be solved.

Proposition 1 presents the solution to problem (1). Firstly, estimate the parameters $\hat{\theta }$ and ${\hat{\gamma }}_{c}$ (1≤c≤C) by minimizing problem (5) on the training data, i.e., minimizing:

$$f(\theta ,\Gamma )≜-\frac{\log p(H_{1:C}|\theta ,\Gamma )}{m}≜-\frac{1}{m}\sum_{c=1}^{C}\sum_{i=1}^{m_{c}}\log p(h_{c}^{\left(i\right)}|\theta ,\gamma_{c}).$$ (7)

The above equation represents the negative log-likelihood function of model (2). Next, utilize (5) to obtain models for the *C* clients involved in training during training time. Lastly, to handle unseen client $c_{new}\notin [C]$ during training, maintain the base models unchanged, select the weights $\gamma_{c_{new}}$ that maximize the likelihood of client data, and predict the local model for the client using (5).

**Lemma 1.** *Under Assumptions 1 and 2, let $\hat{\theta }$ and $\hat{\Gamma }$ be the solution to problem (8), then we have:*

$$p_{c}(x,y|\hat{\theta },{\hat{\gamma }}_{c})=p_{c}(x,y|\theta^{\prime \prime },{\gamma_{c}^{\prime }}^{\prime }),\;\forall c\in C.$$ (8)

**Lemma 2.** *Represent the N probability distributions on Y as *q**_*n*_ *for n∈[N], and let $\alpha =(\alpha_{1},\cdots ,\alpha_{n})\in \Delta^{N}$. For any probability distribution q on Y, if and only if $q=\sum_{n=1}^{N}\alpha_{n}\cdot q_{n}$, it holds that:*

$$\sum_{n=1}^{N}\alpha_{n}\cdot KL(q_{n}∥q)\geq \sum_{n=1}^{N}\alpha_{n}\cdot KL(q_{n}∥\sum_{n^{\prime }=1}^{N}\alpha_{n^{\prime }}\cdot q_{n^{\prime }}).$$ (9)

**Lemma 3.** *Since $\hat{\theta }$ and $\hat{\Gamma }$ are solutions to problem (5), under Assumptions 1, 2, and 3, if *r**_*s*_ *does not depend on s∈S, it is possible to minimize $L_{D_{c}}(s_{c})=E_{(x,y)\sim D_{c}}[l(s_{c}(x),y)]$ by the model $s_{c}^{\prime \prime }(c\in C)$. When (x,y)∈X×Y, it can be proven:*

$$p_{{s_{c}^{\prime }}^{\prime }}(y|x)=\sum_{n=1}^{N}{\hat{\gamma }}_{cn}\cdot p_{n}(y|x,{\hat{\theta }}_{n}).$$ (10)

According to Lemma 3, *p*_*c*_(*x*,*y*)depends on $\theta^{\prime \prime }$and ${\gamma_{c}^{\prime }}^{\prime }$.

For s∈S and (x,y)∈X×Y, let *p*_*s*_(*y*|*x*) denote the conditional probability distribution of *y* given *x* under model *s*, defined as:

$$p_{s}(y|x)≜e^{r_{s}(x)}\;\times \;\exp\{-l(s(x),y)\},$$ (11)

where

$$r_{s}(x)≜-\log\left[\int_{y\in Y}\exp\{-l(s(x),y)\}dy\right].$$ (12)

The entropy of a probability distribution *q* on *Y* can be expressed as:

$$H(q)≜-\int_{y\in Y}q(y)\cdot \log q(y)dy,$$ (13)

the Kullback-Leibler (KL) divergence between two probability distributions *q*_1_ and *q*_2_ on *Y* (a measure of the asymmetry of difference between two distributions) can be represented as:

$$KL(q_{1}∥q_{2})≜\int_{y\in Y}q_{1}(y)\cdot \log\frac{q_{1}(y)}{q_{2}(y)}dy.$$ (14)

When using mean squared error, regression loss, and cross-entropy loss functions, we verified that in these three cases, *r*_*s*_ is independent of *s*, and then concluded using Lemma 3.

**Mean squared error.** This is a regression problem where *g* > 0, and there exists $Y={\mathbb{R}}^{g}$. For x,y∈X×Y, s∈S, we have:

$$p_{s}(y|x)=\frac{1}{\sqrt{(2\pi {)}^{g}}}\cdot \exp\left\{-\frac{\|s(x)-y{\|}^{2}}{2}\right\},$$ (15)

and

$$r_{s}(x)=-\log\left(\sqrt{(2\pi {)}^{g}}\right).$$ (16)

**Regression loss.** This is a binary classification problem where *L* > 1, and *Y* = [*L*]. For x,y∈X×Y, s∈S, we have:

$$p_{s}(y|x)=(s(x){)}^{y}\cdot (1-s(x){)}^{1-y},$$ (17)

and

$$r_{s}(x)=0.$$ (18)

**Cross-entropy loss.** This is a classification problem where *L* > 1, and *Y* = [*L*]. For x,y∈X×Y and s∈S, we have:

$$p_{s}(y|x)=\prod_{l=1}^{L}(s(x){)}^{1_{\left\{y=l\right\}}},$$ (19)

and

$$r_{s}(x)=0.$$ (20)

**Theorem.** *For c∈C, consider a model ${s_{c}^{\prime }}^{\prime }$ that minimizes $E_{(x,y)\sim D_{c}}[l(s_{c}(x)),y]$. Using Lemma 3, for (x,y)∈X×Y, we have:*

$$p_{s_{c}^{\prime \prime }}(y|x)=\sum_{n=1}^{N}{\hat{\gamma }}_{cn}\cdot p_{n}\left(y|x,{\hat{\theta }}_{n}\right).$$ (21)

Multiplying both sides of the equation by *y* and integrating over y∈Y, in all three cases, we have:

$$\forall x\in X,\int_{y\in Y}y\cdot p_{s}(\cdot |x)dy=s(x),$$ (22)

therefore,

$${s_{c}^{\prime }}^{\prime }=\sum_{n=1}^{N}{\hat{\gamma }}_{cn}s_{{\hat{\theta }}_{n}},\forall c\in C.$$ (23)

Meaning the base model can represent any model in the client set *C*. As long as Lemma 1, Lemma 2, and Lemma 3 hold, the existence of the base model can be proven. We have included the proofs of Lemma 1, Lemma 2, and Lemma 3 in the S1 Appendix.

## 4 Solving base model using the evolutionary algorithm

Building on the existence of base models, this section introduces the method for solving base models using evolutionary algorithms and the corresponding algorithmic procedure.

### 4.1 Base model solving

Before delving into the methodology of this paper, it is essential to provide the process of FL and the symbols used. FL is a distributed machine learning framework, which consists of a central server for model aggregation and multiple distributed clients for executing intelligent computing tasks. Assuming there are *N* clients, each client has a data volume of *D*_*i*_(*i* = 1,2,...,*N*), and the model is denoted as $\theta_{i}$. The server does not store data. In each round of iteration, the server first distributes the global model θ(0) to all clients. Subsequently, each client conducts local training based on the received model. The training process is as follows:

$$\theta_{i}(t,e+1)=\theta_{i}(t,e)-\eta \nabla F(\theta_{i}(t,e)),e=0,2,\cdots ,E-1,$$ (24)

where $\theta_{i}(t,e\;+\;1)$ represents the model parameters after the *e*-th local training in the *t*-th global iteration. In each round of iteration, the client conducts *E* local trainings. $F_{i}(•)$ is the loss function of the *i*-th client after local training.

After local training by all clients is completed, the server randomly selects $\tilde{N}$ clients from *N* clients for aggregation. Taking FedAvg as an example, the server aggregates based on the weights of the data volume uploaded by selected clients. The aggregation process is as follows.

$$\theta (t+1)=\sum_{j=1}^{\tilde{N}}\frac{D_{j}}{D}\theta_{j}(t,E),\;t=1,2,\cdots ,T,$$ (25)

where θ(t) represents the global model aggregated in the *t*-th iteration process, *T* is the total number of iterations, and the calculation method of *D* is as follows:

$$D=\sum_{j=1}^{\tilde{N}}D_{j}.$$ (26)

Finally, the server redistributes the global model to the clients for training, and the above process is iterated repeatedly until convergence or the preset number of iterations is reached.

After the description of the classic FL method, we introduce a base model solving approach based on evolutionary algorithms. The primary aim of this method is to reform the client selection process, which traditional approach is to randomly select clients. To enhance aggregation efficiency, this paper proposes using a classic evolutionary algorithm, the genetic algorithm to solve for the base models to be aggregated. This approach involves several key operations, including genetic encoding, crossover, and mutation.

In terms of genetic encoding, the length of the chromosome is set to $\tilde{N}$, where each gene on the chromosome represents the ID of a selected client. There are a total of *N* clients, with client IDs ranging from 0 to (*N*–1). After a certain number of federated iterations, a genetic algorithm is applied to optimize the selected clients, aiming to identify the base models in *N* client models as much as possible. The population size is denoted as *pop*_−_*size*.

In each iteration, 50% of the superior individuals are retained from the parent population. Each chromosome can be represented as $Chm_{j}=[id_{1},id_{2},\cdots ,id_{\tilde{N}}]$, where *j* is the chromosome number (j=1,2,...,pop_size). The chromosomes are not affected by the order of genes, and there are no duplicate genes within a chromosome. Therefore, two chromosomes with different gene orders are considered as the same chromosome.

**Fig 2:**
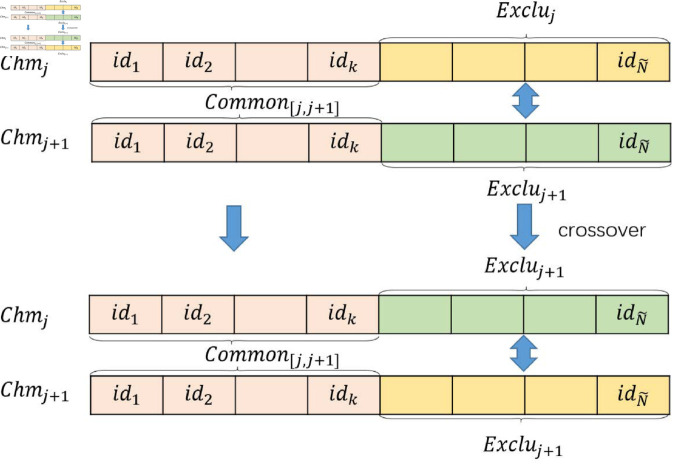
Crossover operator.

**Crossover Operator.** The crossover probability is typically set to 1. For every adjacent pair of chromosomes *Chm*_*j*_ and *Chm*_*j* + 1_, the intersection set *Common*_*[j*,*j* + 1*]*_ is obtained, which consists of the client IDs that are present in both chromosomes. Thus, each chromosome *Chm*_*j*_ is composed of two parts: the common IDs and the unique IDs.

$$Chm_{j}=[Common_{\left[j,j+1\right]},Exclu_{j}],$$ (27)

$$Chm_{j+1}=[Common_{\left[j,j+1\right]},Exclu_{j+1}],$$ (28)

the crossover operator exchanges the unique IDs, excluding *Common*_*[j*,*j* + 1*]*_, from two chromosomes *Chm*_*j*_ and *Chm*_*j* + 1_ to create new individuals (as shown in Fig 2).

$$Chm_{j}=[Common_{\left[j,j+1\right]},Exclu_{j+1}],$$ (29)

$$Chm_{j+1}=[Common_{\left[j,j+1\right]},Exclu_{j}]\cdot$$ (30)

**Fig 3:**
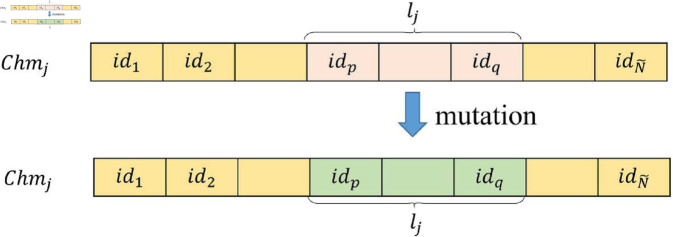
Mutation operator.

**Mutation Operator.** The mutation probability is defined as muta prob, and the mutation length is represented by a random variable *l*_*j*_. A random gene block $Chm_{j}=[id_{1},\cdots ,[\underset{l_{j}}{\underbrace{id_{p},\cdots ,id_{q}}}],\cdots ,id_{\tilde{N}}]$ of length *l*_*j*_ is selected from chromosome *Chm*_*j*_, and the IDs of this gene block are randomly replaced with client IDs that are not present in *Chm*_*j*_ (as shown in Fig 3).

$$loss_{2}=Crossentropy(\hat{y},y),$$ (31)

where Crossentropy ($\hat{y},y$) = $-\sum_{c\in C}{\hat{y}}_{c}\log_{2}y_{c}$.

Finally, the fitness function is obtained as follows:

$$fittness=\frac{1}{loss_{1}+\mu loss_{2}}.$$ (32)

Loss1 aims to minimize the similarity loss of the models in order to select client models that are as orthogonal as possible to the global model, while Loss2 focuses on enhancing classification performance by minimizing cross-entropy. By integrating Loss1 and Loss2, we obtain a fitness function that allows us to simultaneously address feature integration and classification performance during the optimization process. By introducing a hyperparameter *μ*, we can adjust the relative importance of the two losses in the fitness calculation, thereby flexibly adapting to different application scenarios and requirements. Ultimately, we select individuals with high fitness values to meet the selection criteria for client models.

### 4.2 Algorithm

The detailed computation process of the proposed method is described in Algorithm 1. The server initializes the global model and distributes it to individual clients. Each client conducts local training for *E* epochs based on the received global model. After completing the training, the server selects *N* clients for federated aggregation. Every GA iterations, a genetic algorithm is applied to optimize the selected client ID list, aiming to accelerate the convergence speed and effectiveness of the model. This iterative process continues until convergence is reached or the predefined iteration count *T* is achieved.

**Algorithm 1. Efficient Federated Learning via Aggregation of Base Models.**

**Require:** learning rate *η*, federated iteration count *T*, local

training epochs *E*, total number of clients *N*, number of

clients per round $\tilde{N}$, client *i* data $\left(x_{i},y_{i}\right)$ and data size *D*_*i*_,

optimization interval GA_gap

**Ensure:** global model θ(T)

1: Initialize global model θ(0)

2: **for** t=1,2,…,T **do**

3:   Server distributes the initial global model to each client

4:   **for** e=0,1,…,E−1 **do**

5:    $\theta_{i}(t,e+1)=\theta_{i}(t,e)-\eta \nabla F(\theta_{i}(t,e))$

6:   **end for**

7:   **if** t%GA_gap=0 **then**

8:    $Chm_{j}=[id_{1},id_{2},\ldots ,id_{\tilde{N}}]\leftarrow$ Genetic Algorithm optimizes

client IDs

9:    $\theta (t+1)=\sum_{j\in Chm_{j}}\frac{D_{j}}{D}\theta_{j}(t,E)$ //Use optimized client IDs

10:   **else**

11:    Randomly select $\tilde{N}$ client models for aggregation

12:    $\theta (t+1)=\sum_{j=1}^{\tilde{N}}\frac{D_{j}}{D}\theta_{j}(t,E)$

13:   **end if**

14: **end forreturn** global model θ(T)

## 5 Experiment

In order to validate the effectiveness of the proposed method, this section aims to address the following questions through experiments:

- Can using evolutionary algorithms to solve the base model in FL improve the performance of the aggregated model?
- Is the distribution of client data influence the aggregation effect of the proposed method?
- During the process of solving the base model, how do different optimization intervals affect the effectiveness of the aggregated model?

### 5.1 Experiment setting

**Datasets and models.** We extensively evaluate our method on MNIST, FashionMNIST, and TodayNews datasets, and conduct experiments using the LeNet and TextCNN. The MNIST dataset contains 70,000 handwritten digit images, with 60,000 images used for training and 10,000 images for testing. Each image has a size of 28x28 pixels. The dataset consists of 10 classes (digits 0-9), with each class having a relatively uniform sample size of approximately 7,000 images. Similarly, the FashionMNIST dataset also contains 70,000 images, with 60,000 for training and 10,000 for testing, and the images are of the same size (28x28 pixels). This dataset includes 10 categories (such as T-shirts, trousers, shoes, etc.), with a relatively uniform sample size across each category. In both datasets, the sample sizes for each class are relatively uniform. To simulate data heterogeneity among clients in a non-iid scenario, we can partition the datasets using a Dirichlet distribution, resulting in a distribution that more closely resembles real-world applications. The TodayNews dataset contains approximately 30,000 news articles covering various topics. The distribution of categories in this dataset is relatively uneven, with some topics having a large number of articles while others have relatively few. This imbalance makes the TodayNews dataset more suitable for simulating real-world federated learning scenarios.

**Baselines.** We incorporated components of genetic algorithms into the classic federated learning algorithms FedAvg, FedProx, and SCAFFOLD to validate the effectiveness of the components proposed in this paper. We employed the same training settings and dataset allocations across all experiments. The partitioning of the dataset and the allocation of client data were kept consistent to ensure the comparability of experimental conditions. Parameters were adjusted to ensure that all baseline methods achieved optimal performance.

**Hyper-parameters.** The specific parameter settings are shown in Table 1. During the FL process, assuming there are 50 clients, with 10 clients participating in training each round. Based on the description in the literature [58] and considerations regarding computational power, the following points can be made. The population size of the genetic algorithm is set to 100, with a selection probability of 50% and a mutation probability of 10%. Every 10 federated iterations, the genetic algorithm is utilized to optimize the aggregated client IDs.

**Table 1:** Hyper-parameter settings.

Dataset	Learning rate	Optimizer	Batch size
MNIST	0.01	SGD	64
FashionMNIST	0.01	SGD	64
TodayNews	0.01	SGD	64

### 5.2 Independent and identically distributed data

**Fig 4:**
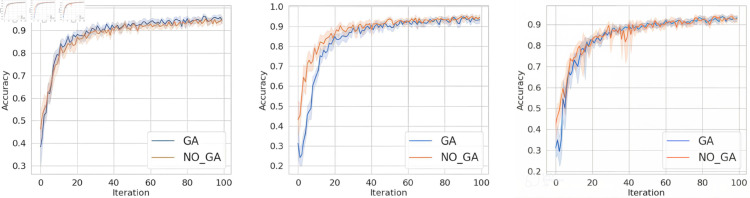
The accuracy of MNIST dataset under FedAvg, FedProx, and SCAFFOLD.

**Fig 5:**
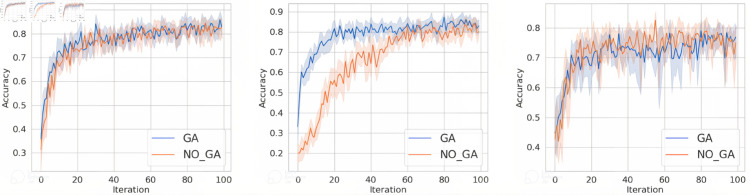
The accuracy of FashionMNIST dataset under FedAvg, FedProx, and SCAFFOLD.

**Fig 6:**
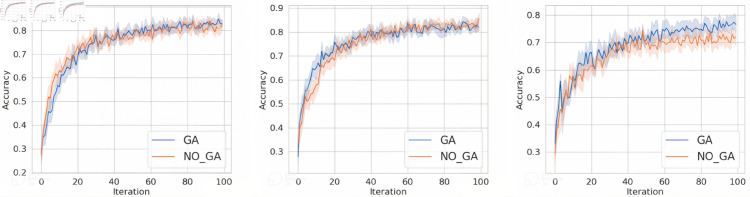
The accuracy of FashionMNIST dataset under FedAvg, FedProx, and SCAFFOLD.

For question 1, conduct experiments under the condition that the client data is independent and identically distributed. This section aims to verify the proposed method in this paper, to investigate whether it can improve the accuracy and convergence speed of the aggregated model compared to not using genetic algorithm optimization. The specific experimental results are shown in Figs 4, 5, and 6. In the figures, the horizontal axis represents the number of training epochs, while the vertical axis indicates the accuracy. The blue line represents the results obtained when using genetic algorithm in the classic FL framework, while the orange line corresponds to the results obtained when training in the classic FL framework without genetic algorithm.

From Figs 4, 5, and 6, it can be observed that when training on the MNIST dataset, FashionMNIST dataset, and TodayNews dataset, under the FedAvg, FedProx, and SCAFFOLD frameworks, incorporating genetic algorithm optimization every 10 iterations leads to faster convergence speed and higher final convergence values (accuracy) compared to not using genetic algorithm. Specifically, our proposed method consistently converges and achieves higher accuracy values in the range of 20-40 iterations. This is attributed to the ability of our method to help the server select base models with larger discrepancies, thereby enhancing the diversity of features fused in the aggregation model. In the case of independent and identically distributed (IID) data, the data distribution is the same across all clients. However, there are still some discrepancies in training performance. The client models selected using the genetic algorithm, which are as orthogonal as possible to the global model, have certain advantages over those obtained through random selection. Therefore, this method demonstrates improved performance compared to random aggregation in federated learning.

### 5.3 Non-independent and identically distributed data

However, in real-world scenarios, due to the varying environments each client faces, the data from clients often exhibit non-independent and identically distributed characteristics.

Therefore, to answer question 2, this section extends the experiments from Sect 5.2 to verify the performance under Non-IID environment, while keeping other settings unchanged. Using the Dirichlet method to partitioning data, with an imbalance factor set to 0.1.

**Fig 7:**
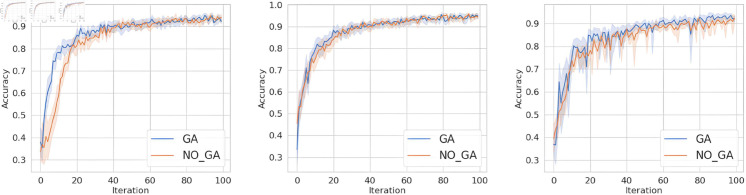
The accuracy of MNIST dataset under FedAvg, FedProx, and SCAFFOLD.

**Fig 8:**
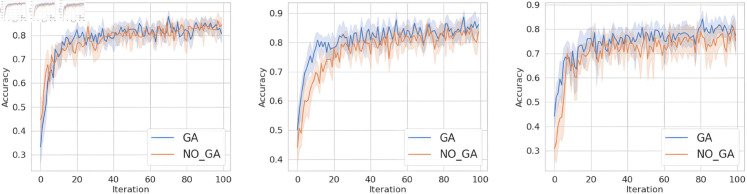
The accuracy of FashionMNIST dataset under FedAvg, FedProx, and SCAFFOLD.

**Fig 9:**
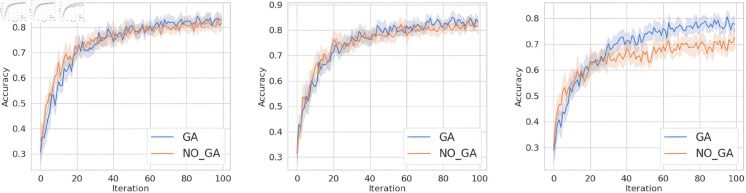
The accuracy of TodayNews dataset under FedAvg, FedProx, and SCAFFOLD.

From Figs 7, 8, and 9, it can be observed that when the data distribution is imbalanced, the advantages of our method become more pronounced compared to the IID scenario in Sect 5.2. In terms of convergence speed, our proposed method consistently tends to converge when the number of iterations is between 20-40, while achieving higher accuracy. This is because the primary issue addressed in this paper is that, under Non-IID settings, the client models selected for aggregation by the server are likely to come from the same (or similar) data distributions, resulting in a biased global model. In the case of non-independent and identically distributed (non-IID) data, the method proposed in this paper can filter client models that are orthogonal to the global model parameters for aggregation, facilitating earlier convergence of the global model. In contrast, the random selection method requires multiple rounds of filtering and aggregation to ensure that the global model covers the data distribution of all client models, resulting in slower convergence of the global model.

To further validate the effectiveness of the proposed method under different degrees of data heterogeneity, we present a comparative performance analysis in the table for algorithms with and without evolutionary components, show the performance comparison when the *α* value is set to 0.5.

Based on the data in the Table 2, the federated learning methods with EA components consistently demonstrate superior performance. They achieve higher accuracy compared to methods without EA, and they also converge more quickly in terms of the number of rounds and the time required. The genetic algorithm takes approximately 46.96 seconds to perform model selection on the server side, we can observe that the use of the EA algorithm reduces the time required for client models to achieve local convergence. Although the EA algorithm increases resource consumption on the server side, it also cuts down on resource use on the client side, thereby optimizing the usability of federated learning methods on resource-constrained end devices.

**Table 2:** Comparison between methods with EA and methods without EA.

Datasets	Methods	Accuracy(%)	rounds	client runtime(s)
MNIST	FedAvg	90.07	55	88.59
	**FedAvg+EA**	**90.57**	**39**	**46.03**
	FedProx	90.64	52	19.31
	**FedProx+EA**	**91.21**	**52**	**19.87**
	SCAFFOLD	90.86	30	18.98
	**SCAFFOLD+EA**	**91.36**	**28**	**11.86**
FashionMNIST	FedAvg	73.73	44	86.56
	**FedAvg+EA**	**74.49**	**22**	**40.50**
	FedProx	69.29	35	69.42
	**FedProx+EA**	**70.54**	**30**	**56.04**
	SCAFFOLD	77.2	16	30.08
	**SCAFFOLD+EA**	**77.99**	**13**	**23.44**
TodayNews	FedAvg	82.08	47	348.39
	**FedAvg+EA**	**86.62**	**43**	**318.25**
	FedProx	75.85	26	209.12
	**FedProx+EA**	**78.52**	**23**	**173.94**
	SCAFFOLD	77.98	24	162.98
	**SCAFFOLD+EA**	**79.53**	**21**	**144.04**

For the case when the *α* is set to 0.5, we conducted a statistical significance test to demonstrate the effectiveness of the proposed method.

**Null Hypothesis (*H***_**0**_**):** There is no significant difference in accuracy between the models with and without the components ($\mu_{\text{with}}-\mu_{\text{without}}=0$).

**Alternative Hypothesis (*H***_**1**_**):** The accuracy of the models with the components is significantly higher than that of the models without the components ($\mu_{\text{with}}-\mu_{\text{without}}>0$).

The calculated *p*–*value* is approximately 0.0015, which is less than 0.05. Therefore, we conclude that, at the 0.05 significance level, there is sufficient statistical evidence to indicate that the inclusion of the components significantly improves accuracy.

### 5.4 Supplementary experiments

#### 5.4.1 Impact of the hyperparameter μ.

In the equation (32), *μ* denotes the balance between diversity and accuracy in the loss function. We’ve designed a series of experiments to identify the optimal value of *μ*. We performed experiments on the MNIST dataset to explore the optimal *μ* value. As shown in the Table 3, we set *μ* to 0.05, 0.2, 0.4, 0.5, and 1. The global model converged fastest and achieved the best performance when *μ* value was in the range of 0.2 to 0.4.

**Table 3:** Impact of the hyperparameter *μ.*

	μ=0.05	μ=0.2	μ=0.4	μ=0.5	μ=1
Epoch	25	20	18	29	31
Acc	71.00	71.43	70.93	71.71	73.00

#### 5.4.2 Impact of different optimization intervals.

After a certain number of federated iterations, the method in this paper utilizes a genetic algorithm to optimize the selected client IDs.

To answer question 3, this section will primarily investigate the impact of the interval size used in genetic algorithm on learning outcomes. In Sects 5.2 and 5.3, Set the interval hyperparameter *GA_gap* to 10. In this section, considering the Non-IID data distribution scenario, different interval sizes are explored to observe the convergence behavior of the algorithm, and investigate the influence of the interval hyperparameter on the federated aggregation model.

**Fig 10:**
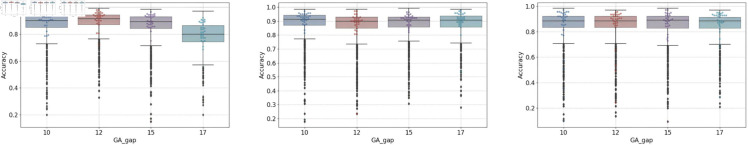
Comparison of MNIST with different intervals under FedAvg, FedProx and SCAFFOLD.

**Fig 11:**
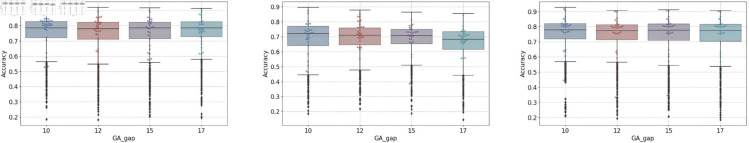
Comparison of TodayNew with different intervals under FedAvg, FedProx and SCAFFOLD.

**Fig 12:**
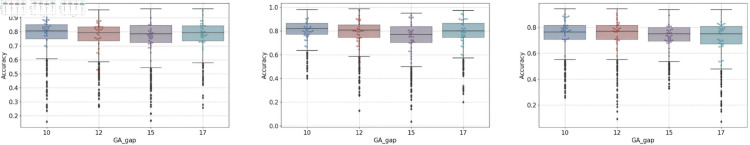
Comparison of FashionMNIST with different intervals under FedAvg, FedProx and SCAFFOLD.

In the experiments, the MNIST, TodayNews, and FashionMNSIT datasets are trained under the genetic algorithm-based FL framework. Furthermore, the interval is increased to 12, 15, and 17, and the results are shown in Figs 10, 11, and 12.

It can be observed from the figures that with a larger *GA_gap*, the optimization rounds for aggregating client models using genetic algorithm are reduced within a limited number of iterations, leading to a slightly decreasing trend in the performance of the final aggregated model. Considering the three methods FedAvg, FedProx, and SCAFFOLD, overall performance is best when *GA_gap*=10.

#### 5.4.3 Impact of the dirichlet distribution.

MNIST experiments with varying Dirichlet distributions to verify our method’s effectiveness under different data heterogeneity levels. As shown in the Table 4, when different data heterogeneity levels are set, the genetic algorithm consistently converges faster than without it, demonstrating the adaptability of our approach across varying heterogeneity levels.

**Table 4:** Impact of the Dirichlet distribution.

Method	α=0.01		α=0.1		α=0.3		α=0.5		α=1	
	EA	NO_EA	EA	NO_EA	EA	NO_EA	EA	NO_EA	EA	NO_EA
FedAvg	**58**	71	**46**	58	**44**	58	**39**	55	**52**	54
FedProx	**44**	58	**42**	47	**46**	52	**52**	52	**39**	50
ScafFold	**27**	28	**21**	23	**22**	29	**28**	30	**19**	25

#### 5.4.4 The analysis of base models.

**Fig 13:**
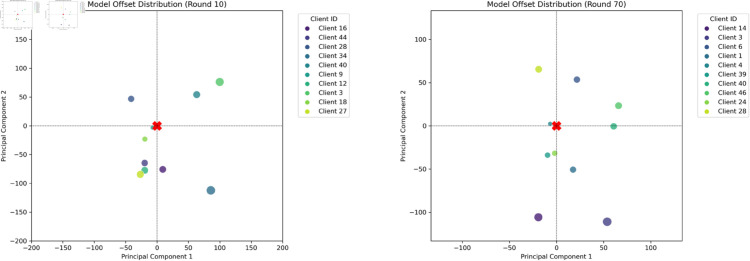
Visualization Comparison Between Client Model with NO_EA.

**Fig 14:**
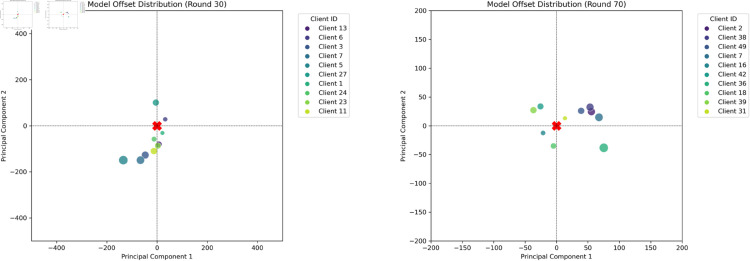
Visualization Comparison Between Client Model with EA.

We use PCA-based dimensionality reduction to visualize the base model’s embedding distribution. The visualizations clearly illustrate the base model’s feature space distribution, further confirming their ability to represent broader model diversity. As shown in the Figs 13 and 14, the client models selected through genetic algorithm have a greater distance between them. The point size represents the distance from the global model, and horizontal and vertical coordinates represent the offset on the first and second principal components after PCA dimensionality reduction. Compared with those selected at random, aggregating base models with larger differences can make the global model converge more rapidly.

The cosine distance is used to calculate the distance between a base model and a global model. The cosine distance is calculated as follows:

$$\text{cosine distance}=1-\frac{A\cdot B}{\left\|A\|\|B\right\|}.$$ (33)

Among them, *A* and *B* are the feature vectors processed by the global model and the client model. The value range of cosine distance is between 0 and 2. The larger the cosine distance, the greater the difference in vector direction. As shown in the Table 5, the base models selected by GA show a greater distance from the globally initialized model. This indicates that our screening approach, while preserving model accuracy, favors the selection of client models with more substantial updates for aggregation. Thereby, it incorporates a wider diversity of client data characteristics, which enhances the convergence of the global model.

**Table 5:** Comparison of model distances.

	EA	NO_EA
MNIST	**0.0136**	0.0082
FashionMNIST	**0.0046**	0.0023
TodayNews	**0.3285**	0.2555

## 6 Discussion

We validated our approach using convolutional neural networks on image and text datasets, including MNIST, FashionMNIST, and TodayNews, achieving encouraging experimental results. Due to limitations in computational resources and time, we did not conduct further validation on larger-scale datasets or networks. To enhance the generalizability of our research, we plan to extend our work to larger datasets in the future to comprehensively evaluate the model’s performance and stability. Additionally, testing the adaptability of different network architectures will be a key focus of our subsequent research to ensure that the model maintains consistent performance across a wider range of applications. Through these expansions, we aim to further validate our conclusions and lay the groundwork for future studies.

**Limitation.** Using the evolutionary algorithm to select clients that are as orthogonal as possible reduces the number of iterations, but it increases the computational consumption on the server during the server’s preference process, even though we typically assume that the server’s computing resources are unlimited. Frequent combination and exchange of client models on the server side can intrinsically pose a risk of model privacy leakage. In future work, we will explore the use of differential privacy and other techniques to better protect client - side data security during model transmission.

## 7 Conclusion

This paper theoretically proves the existence of base models in the FL framework, and proposes an efficient federated learning method via aggregation of base models. This method utilizes the evolutionary algorithm to derive base models from clients with diverse data features, aiming to integrate data characteristics from different distributions in the global model as much as possible. It effectively addresses the issue of biased aggregation results from randomly selected clients in FL, thus accelerating the convergence speed and improving the effectiveness of federated aggregation. Using datasets such as MNIST, FashionMNIST, and TodayNews, and frameworks like FedAvg, FedProx, and SCAFFOLD, it is validated that under IID or Non-IID environment, the global model obtained through the aggregation of base models proposed in this paper, achieves faster convergence and better performance in FL.

## Supporting information

S1 Appendix Proof of Lemmas. (PDF)
