# Supplementary material for: Efficient federated learning via aggregation of base models
Source: PLoS One. 2025 Aug 14;20(8):e0327883. doi: 10.1371/journal.pone.0327883 (PMC12352788; doi:10.1371/journal.pone.0327883)
Supplement: S1 Appendix — (PDF) [file pone.0327883.s001.pdf]

S1 Appendix

Proof of Lemmas 1, 2, and 3 in this section provide theoretical support for the existence of the base models, ensuring the feasibility of the proposed algorithm in this paper.

Proof of Lemma 1. *Based on Assumption 1 and Assumption 3, the following formula holds:*

$$p_c(x, y \mid \hat{\theta}, \hat{\gamma}_c) = p_c(x, y \mid \theta'', \gamma''_c), \forall c \in C. \tag{1}$$

Proof process is as follows.

$$\begin{aligned}
&\underset{(x,y)\sim D_c}{\mathrm{E}}\left[-\log p_c(x, y|\hat{\theta}, \hat{\gamma}_c)\right] \\
&= -\int_{(x,y)\in X\times Y} p_c(x, y \mid \theta'', \gamma''_c) \cdot \log p_c(x, y \mid \hat{\theta}, \hat{\gamma}_c) dxdy \\
&= -\int_{(x,y)\in X\times Y} p_c(x, y \mid \theta'', \gamma''_c) \cdot \log \frac{p_c(x, y \mid \hat{\theta}, \hat{\gamma}_c)}{p_c(x, y \mid \theta'', \gamma''_c)} dxdy \\
&- \int_{(x,y)\in X\times Y} p_c(x, y \mid \theta'', \gamma''_c) \cdot \log p_c(x, y \mid \theta'', \gamma'') dxdy \\
&= KL\left(p_c(\cdot \mid \theta'', \gamma''_c) \parallel p_c(\cdot \mid \hat{\theta}, \hat{\gamma}_c)\right) + S[p_c(\cdot \mid \theta'', \gamma''_c)].
\end{aligned} \tag{2}$$

Since the KL divergence is non-negative, it follows that:

$$\begin{aligned}
S[p_c(\cdot \mid \theta'', \gamma''_c)] = \underset{(x,y)\sim D_c}{\mathrm{E}}[-\log p_c(x, y \mid \theta'', \gamma''_c)] \leq \\
\underset{(x,y)\sim D_c}{\mathrm{E}}\left[-\log p_c(x, y \mid \hat{\theta}, \hat{\gamma}_c)\right].
\end{aligned} \tag{3}$$

Taking the expectation over $c \sim D_c$, we obtain:

$$\begin{aligned}
&\underset{c\sim D_C}{\mathrm{E}}\ \underset{(x,y)\sim D_c}{\mathrm{E}}[-\log p_c(x, y|\hat{\theta}, \hat{\gamma}_c)] \\
&\quad \geq \underset{c\sim D_C}{\mathrm{E}}\ \underset{(x,y)\sim D_c}{\mathrm{E}}[-\log p_c(x, y|\theta'', \gamma''_c)],
\end{aligned} \tag{4}$$

furthermore, we have:

$$\begin{aligned}
\underset{c\sim D_C}{\mathrm{E}}\ \underset{(x,y)\sim D_c}{\mathrm{E}}[-\log p_c(x, y|\hat{\theta}, \hat{\gamma}_c)] \leq \\
\underset{c\sim D_C}{\mathrm{E}}\ \underset{(x,y)\sim D_c}{\mathrm{E}}[-\log p_c(x, y|\theta'', \gamma''_c)].
\end{aligned} \tag{5}$$

Combining (35), (36), and (37), we obtain:

$$\underset{c\sim D_C}{\mathrm{E}} KL\Big(p_c(\cdot \mid \theta'', \gamma'') \parallel p_c(\cdot \mid \hat{\theta}, \hat{\gamma}_c)\Big) = 0. \tag{6}$$

Since the KL divergence is non-negative and D_c is a data distribution on a countable set C, we have:

$$\forall c \in C, KL\Big(p_c(\cdot|\theta'', \gamma''_c) \parallel p_c(\cdot|\hat{\theta}, \hat{\gamma}_c)\Big) = 0, \tag{7}$$

therefore, we can obtain:

$$p_c(x, y|\hat{\theta}, \hat{\gamma}_c) = p_c(x, y|\theta'', \gamma''_c), \forall c \in C. \tag{8}$$

$$p_c(x, y \mid \hat{\theta}, \hat{\gamma}_c) = p_c(x, y \mid \theta'', \gamma_c''), \forall c \in C. \tag{9}$$

Proof of Lemma 2. *Representing N probability distributions on Y as q_n, $n \in [N]$, where $\alpha = (\alpha_1, \cdots, \alpha_n) \in \Delta^N$, for any probability distribution q on Y, $q = \sum_{n=1}^{N} \alpha_n \cdot q_n$, if and only if, we obtain:*

$$\sum_{n=1}^{N} \alpha_n \cdot KL(q_n \parallel q) \geq \sum_{n=1}^{N} \alpha_n \cdot KL\Bigg(q_n \parallel \sum_{n'=1}^{N} \alpha_{n'} \cdot q_{n'}\Bigg). \tag{10}$$

The proof process is as follows.

$$\begin{aligned}
&\sum_{n=1}^{N} \alpha_n \cdot KL(q_n \parallel q) - \sum_{n=1}^{N} \alpha_n \cdot KL\Bigg(q_n \parallel \sum_{n'=1}^{N} \alpha_{n'} \cdot q_{n'}\Bigg) \\
&= \sum_{n=1}^{N} \alpha_n \cdot \left[KL(q_n \parallel q) - KL\left(q_n \parallel \sum_{n'=1}^{N} \alpha_{n'} \cdot q_{n'}\right)\right] \\
&= -\sum_{n=1}^{N} \alpha_n \int_{y \in Y} q_n(y) \cdot \log\left(\frac{q(y)}{\sum_{n'=1}^{N} \alpha_{n'} \cdot q_{n'}(y)}\right) \\
&= -\int_{y \in Y} \left\{\sum_{n=1}^{N} \alpha_n \cdot q_n(y)\right\} \cdot \log\left(\frac{q(y)}{\sum_{n'=1}^{N} \alpha_{n'} \cdot q_{n'}(y)}\right) dy \\
&= KL\left(\sum_{n=1}^{N} \alpha_n \cdot q_n \parallel q\right) \geq 0.
\end{aligned} \tag{11}$$

Proof of Lemma 3 *Considering that $\hat{\theta}$ and $\hat{\Gamma}$ are solutions to problem (8), under Assumptions 1, 2, and 3, if r_s does not depend on $s \in S$, then by minimizing the predictor $s_c''(c \in C)$ to minimize $L_{D_c}(s_c) = E_{(x,y)\sim D_c}[l(s_c(x), y)]$* when $(x, y) \in X \times Y$, it can be proven that:

$$p_{s_c''}(y \mid x) = \sum_{n=1}^{N} \hat{\gamma}_{cn} \cdot p_n(y \mid x, \hat{\theta}_n). \tag{12}$$

The proof process is as follows.

For $c \in C$ and $s_c \in S$, under Assumptions 1, 2, and 3, it holds that:

$$\mathop{\mathrm{E}}_{(x,y)\sim D_c}[l(s_c(x), y)] = int_{x,y \in X \times Y} l(s_c(x) \cdot p_c(x, y \mid \theta'', \gamma_c''))dxdy. \tag{13}$$

By utilizing Lemma 1, it can be derived that:

$$E_{(x,y)\sim D_c}[l(s_c(x), y)] = \int_{x,y \in X \times Y} l(s_c(x) \cdot p_c(x, y \mid \hat{\theta}, \hat{\gamma}_c))dxdy. \tag{14}$$

Substituting Assumptions 1 and 2 into (35), we can obtain:

$$
\begin{aligned}
& \underset{(x,y)\sim D_c}{\mathrm{E}}[l(s_c(x),y)] \\
&= \int_{x,y\in X\times Y} l(s_c(x),y)\cdot p_c\Big(x,y\mid\hat{\theta},\hat{\gamma}_c\Big)dxdy \\
&= \int_{x\in X}\left[\sum_{n=1}^{N}\hat{\gamma}_{cn}\right]_{y\in Y} l(s_c(x),y)\cdot p_n\Big(y|x,\hat{\theta}_n\Big)dy\Bigg]p(x)dx \\
&= \int_{x\in X}\left[\sum_{n=1}^{N}\hat{\gamma}_{cn}\Big\{r_{s_c}(x)-\int_{y\in Y}p_n\left(y\mid x,\hat{\theta}_n\right)\log p_{s_c}(y\mid x)dy\Big\}\right]p(x)dx \\
&= \int_{x\in X}\left[r_{s_c}(x)-\sum_{n=1}^{N}\hat{\gamma}_{cn}\int_{y\in Y}p_n\left(y\mid x,\hat{\theta}_n\right)\log p_{s_c}(y\mid x)dy\right]p(x)dx \\
&= \int_{x\in X}\left[r_{s_c}(x)+\sum_{n=1}^{N}\hat{\gamma}_{cn}\cdot S\Big(p_n\Big(\cdot|x,\hat{\theta}_n\Big)\Big)\right]p(x)dx \\
&+ \int_{x\in X}\left[\sum_{n=1}^{N}\hat{\gamma}_{cn}\cdot KL\Big(p_n\Big(\cdot\mid x,\hat{\theta}_n\Big)\parallel p_{s_c}(\cdot\mid x)\Big)\right]p(x)dx.
\end{aligned}
\tag{15}
$$

Let s'_c be a model that satisfies the following equation:

$$
p_{s'_c}(y\mid x)=\sum_{n=1}^{N}p_n\left(y\mid x,\hat{\theta}_n\right)\cdot\hat{\gamma}_{cn}, \tag{16}
$$

by utilizing Lemma 2, if and only if:

$$
p_{s_c}(\cdot\mid x,\tilde{\theta}_n)=p_{s'_c}(\cdot\mid x), \tag{17}
$$

this leads to:

$$
\sum_{n=1}^{N}KL\Big(p_n(\cdot|x,\hat{\theta}_n)\parallel p_{s_c}(\cdot|x)\Big)\cdot\hat{\gamma}_{cn}\geq\sum_{n=1}^{N}KL\Big(p_n(\cdot\mid x,\hat{\theta}_n)\parallel p_{s'_c}(\cdot\mid x)\Big)\cdot\hat{\gamma}_{cn}. \tag{18}
$$

Since r_s does not depend on s, substituting equation (40) into equation (47), we obtain:

$$
E_{(x,y)\sim D_c}[l(s'_c(x),y)]\leq E_{(x,y)\sim D_c}[l(s_c(x),y)]. \tag{19}
$$

The above inequality holds for any model s_c; therefore, it also applies to the model $s''_c\in\arg\min\mathrm{E}_{(x,y)\sim D_c}[l(s_c(x),y)]$.

In conclusion, Lemma 1, Lemma 2, and Lemma 3 have been proven. Consequently, in the framework of FL, there exists base models can represent all client models in this framework through the parameters average combination.
